# Prior resilience to trauma & coping during the COVID-19 pandemic

**DOI:** 10.1371/journal.pone.0297169

**Published:** 2024-05-07

**Authors:** Arielle A. J. Scoglio, Kristen Nishimi, Karmel W. Choi, Karestan C. Koenen, Laura A. Sampson, Shaili C. Jha, Laura D. Kubzansky

**Affiliations:** 1 Department of Epidemiology, Harvard T.H. Chan School of Public Health, Boston, Massachusetts, United States of America; 2 Department of Natural and Applied Sciences, Bentley University, Waltham, Massachusetts, United States of America; 3 Mental Health Service, San Francisco Veterans Affairs Medical Center, San Francisco, California, United States of America; 4 Department of Psychiatry, Massachusetts General Hospital, Boston, Massachusetts, United States of America; 5 Department of Social and Behavioral Sciences, Harvard T.H. Chan School of Public Health, Boston, Massachusetts, United States of America; The John Paul II Catholic University of Lublin, POLAND

## Abstract

**Background and objective:**

This study examined the potential influence of pre-pandemic psychological resilience on use of approach or avoidant coping styles and strategies to manage stress during the COVID-19 pandemic. We hypothesized that higher resilience would be associated with more approach coping and less avoidant coping.

**Design and methods:**

Longitudinal cohort data were from the Nurses’ Health Study II, including 13,143 female current and former healthcare professionals with pre-pandemic lifetime trauma. Pre-pandemic resilience was assessed between 2018–2019 and current coping during the outbreak of the pandemic in the United States (May-August 2020). Multiple linear regression model results identified associations between continuous pre-pandemic resilience scores and use of approach and avoidant coping styles, as well as individual coping strategies, adjusting for relevant covariates.

**Results:**

Greater resilience was associated with higher use of approach coping (ß = 0.06, 95% CI 0.05, 0.08) and lower use of avoidant coping styles (ß = -0.39, 95% CI -0.41, -0.38). Higher pre-pandemic resilience was also associated with use of eight (distraction [ß = -0.18, 95% CI -0.20, -0.16], substance use [ß = -0.15, 95% CI -0.17, -0.13], behavioral disengagement [ß = -0.29, 95% CI -0.30, -0.27], self-blame [ß = -0.44, 95% CI -0.45, -0.42], emotional support (ß = 0.03, 95% CI 0.01, 0.05), positive reframing [ß = 0.13, 95% CI 0.12, 0.15], humor [ß = 0.03, 95% CI 0.01, 0.05] and religion [ß = 0.06, 95% CI 0.04, 0.08]) of the nine coping strategies in expected directions.

**Conclusion:**

Findings have important implications for intervention or even prevention efforts to support vulnerable groups, such as women with prior trauma histories, during this and other immensely stressful times. Supporting or building psychological resilience following trauma may promote effective coping in times of future stress.

## Introduction

Coping can be defined as the use of cognitive and/or behavioral strategies to manage a taxing internal or external situation [1]. The COVID-19 pandemic has been a globally stressful experience which has brought losses and major life changes for many people, such as financial hardship, job loss, social isolation, and/or family illness [2]. Previous research suggests that effective coping may guard against the development of psychiatric or other disorders during highly stressful events such as natural disasters [3]. Whether prior psychological resilience to trauma predicts coping in the context of a new traumatic or stressful event (e.g., COVID-19 pandemic) warrants further study. Resilience to trauma can be understood as positive psychological adaptation (e.g., evidenced by the presence of well-being and/or the absence of psychological distress) in the context of past exposure to traumatic events.

Some research has examined specifically how coping strategies are associated with mental health during infectious disease epidemics and reported associations between avoidant coping strategies and poorer mental health in studies using both cross-sectional and longitudinal designs (Kar et al., 2021; Main et al., 2011). This implies there may be a bidirectional relationship between mental health and coping. For example, among Chinese undergraduate students experiencing the 2003 SARS pandemic, avoidant forms of coping were associated with higher anxiety, depression, and somatization [4,5] both cross-sectionally and longitudinally, before the SARS outbreak and 5 months later. In contrast, other coping strategies, such as humor and seeking emotional support from others, were cross-sectionally associated with fewer mental health problems (e.g., depression, anxiety) during the COVID-19 pandemic [6,7]. As the COVID-19 pandemic is an unprecedented global catastrophe, a more detailed examination of coping strategies during this time using longitudinal data, especially among groups that may be at particularly high risk for negative mental health outcomes (e.g., individuals with prior trauma exposures), is important to inform prevention and intervention efforts.

### Approach and avoidant coping styles

While individuals may use a variety of coping strategies to manage stressors, prior literature suggests that certain combinations of similar coping strategies may denote an overall coping style. An approach coping style involves efforts to find solutions, understand causes, and accept the presence of a stressor or problem. In contrast, an avoidant coping style involves efforts to ignore, disengage, or distract oneself from a stressor or problem [8]. Traditionally, an approach coping style, and the strategies that make up this style (active coping, seeking emotional support, positive reframing) are considered more adaptive means of coping with stressors, while an avoidant coping style and its strategies (substance use, self-blame, self-distraction, behavioral disengagement) are considered less adaptive in the face of stress. It is important to note that what are considered “effective” coping strategies are dependent on context and specific strategies may not be consistently effective across stressful situations [7]. For example, strategies such as humor or religion may be more or less adaptive depending on the context. Therefore, the flexibility to use different coping strategies depending on the context may be most adaptive [9]. However, in prior literature, an approach coping style has been associated with more adaptive responses to adversity, better physical health, and higher psychological well-being, while an avoidant coping style has been associated with subsequent poorer physical health among those with medical conditions, and poorer mental health outcomes such as anxiety [10,11].

### Connecting trauma, resilience and coping

Traumatic events may lead to the development of an avoidant coping style or increase the likelihood of using avoidant coping strategies. For example, prior work has found individuals with a history of childhood abuse, intimate partner violence, or adult sexual assault are more likely to demonstrate an avoidant coping style through the use of strategies viewed as less adaptive (e.g., substance use; [12–14]. In a Mechanical Turk study of 674 individuals, avoidance coping predicted worse mental health adjustment to the pandemic [15]. As prior evidence suggests that trauma exposure, subsequent distress, and avoidant coping are related, it is possible that individuals who are psychologically resilient following trauma exposure may demonstrate more approach coping. Emerging evidence during the pandemic has also shown that teaching approach coping skills may strengthen psychological health in the face of COVID-related stress [16–18] for healthcare professionals.

Conceptualizations and operationalizations of resilience are varied in prior literature. Luthar, Cicchetti and Becker [19] define the construct as “a dynamic process encompassing positive adaptation within the context of significant adversity” and build this definition based on prior work which recognizes that resilience can be measured when there has been exposure to adversity and achievement of positive adaptation despite hardship [20,21]. Bonanno [22] posits specifically that resilience is a dynamic process that manifests as a stable trajectory of mental health in the aftermath of a severe stressor. In contrast, other scholars conceptualize resilience as an individual trait [23] Given heterogeneity in conceptualizations of resilience, and ambiguity in definitions and utility of the construct, it is important for studies of resilience to indicate clearly the theoretical approach underpinning their work [19] In this study, we define resilience as a manifested outcome reflecting one’s level of psychological functioning or adaptation following trauma exposure, following the work of Choi and colleagues [24]. Using this conceptual definition of manifested resilience, we incorporated several existing frameworks to understand how resilience would be related to stress, mental health, and coping. More specifically, we draw on two distinct but complementary theoretical frameworks to connect resilience to trauma and coping styles and strategies: positive appraisal style theory of resilience (PASTOR) and the integrative affect-regulation framework for resilience. PASTOR posits that through positive appraisal and subsequent reappraisal processes, resilience factors converge and exert protective effects on mental health [25]. Related, the integrative affect-regulation framework for resilience combines insights regarding stress and coping with those derived from work on emotion regulation [26]. This framework suggests an individual’s cognitive appraisals of stressors and the strategies used to regulate emotion when confronted by stressors together drive subsequent behavioral and psychological responses (i.e. coping behaviors, mental health symptoms; [26]. Both perspectives are relevant for considering whether and how resilience to trauma might affect downstream behaviors as well as psychological, and biological processes. Exposure to trauma can disrupt capacity to regulate emotion effectively and thereby increase risk for developing mental health problems. These disruptions in turn can affect strategies individuals use to cope with subsequent stressors [27]. Both theoretical perspectives posit that trauma disrupts effective emotion regulation or appraisals of current and subsequent stressors, in ways that increase risk of developing mental health problems. In contrast, effective emotion regulation and positive appraisals following trauma may lead to resilience and ultimately more favorable psychological health than would be expected given the trauma experienced. Resilience is therefore a complex and multidimensional construct that involves positive and adaptive elements of wellbeing and an absence of psychological distress [28,29] following traumatic experiences.

A universal stressor such as the COVID-19 pandemic affords a unique opportunity to examine how prior resilience to trauma may promote positive coping during a time of collective stress. In a prior study (Choi et al., 2022), pre-pandemic psychological resilience to lifetime trauma was associated with better mental health outcomes during the first months of the pandemic, including higher subjective well-being and lower risk of depression, anxiety, and posttraumatic stress. Because the stress-sensitization model posits that trauma-exposed individuals are at an elevated risk for poor mental health outcomes when confronted with subsequent stressors [30,31], these findings extend the model by exploring how levels of psychological functioning following trauma, indicating responses ranging from less to more resilient, may influence how one faces subsequent stressors. Individuals showing higher resilience to prior trauma may be more likely to engage in adaptive coping in response to future stress, which may in turn result in more favorable mental health. Although the COVID-19 pandemic can be understood as a collective traumatic experience, negative effects of cumulative trauma are well documented. Thus, individuals who showed prior resilience may use adaptive coping strategies in the face of stress in the context of the COVID-19 pandemic, and perhaps cope in different ways than individuals with relatively lower resilience. For example, psychotherapy studies have suggested that promoting adaptive management of stressful experiences may help to inoculate against distress to later stressors [32]. However, the role of prior resilience in shaping coping behavior during a major stressor such as the COVID-19 pandemic is not well understood.

An important and unique additional dimension to consider in the context of the COVID-19 pandemic may be healthcare professional status. Healthcare professionals may learn coping skills during their training and could be better equipped to deal with such a serious stressor than individuals working in other fields. On the other hand, healthcare professionals during the pandemic have been bombarded with chronic severe stress at work and may feel overloaded [18]. Doctors, nurses, and others providing care during the pandemic have had to confront the challenges of the pandemic in different ways from the general population, and therefore, may have different patterns of coping or their coping levels may exhibit a differential association with resilience. Our present study affords a unique opportunity to examine resilience levels and coping strategies used by a cohort of current and former healthcare professionals.

### Present study

**Fig 1:**
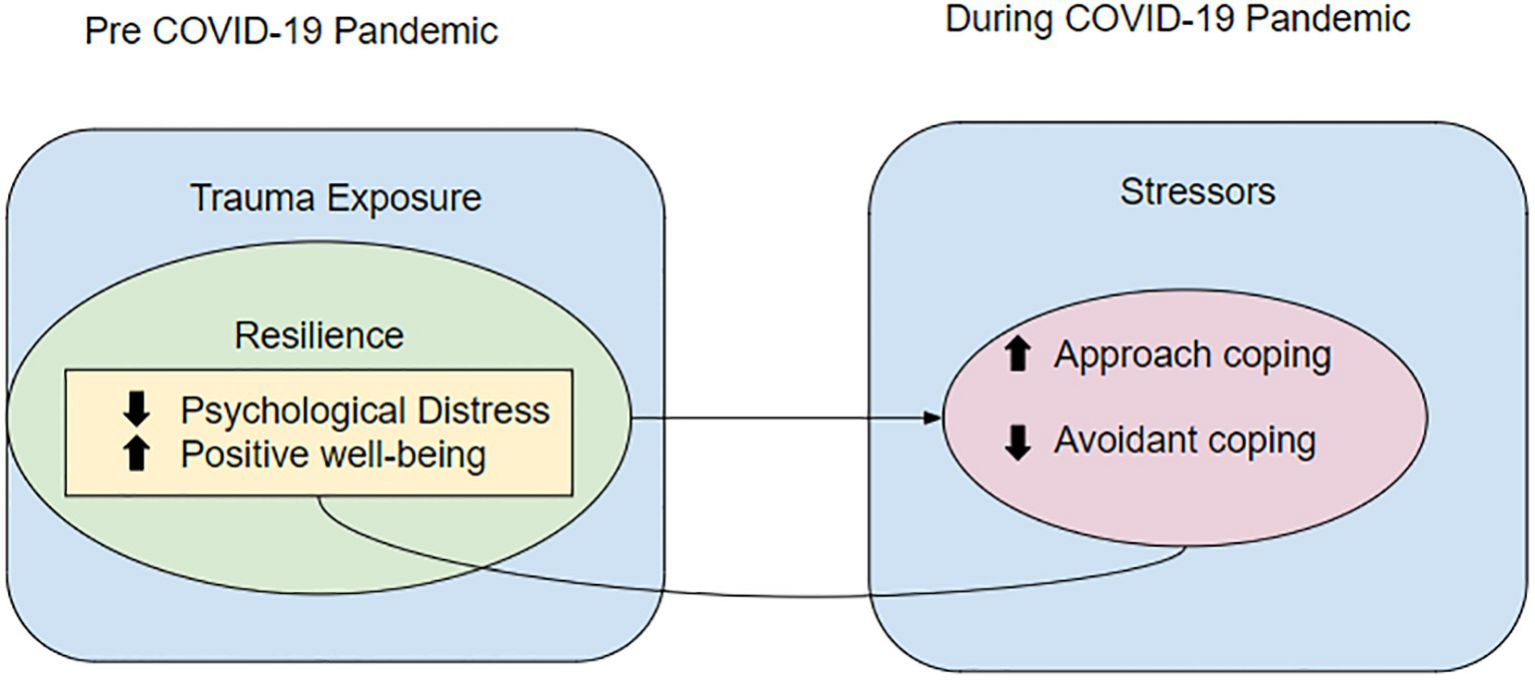
Adapted conceptual framework. Note: Dotted line denotes a theorized feedback loop between coping and psychological health.

Drawing on the aforementioned theories, we have developed an adapted conceptual framework for this study represented in Fig 1. In our adapted framework, pre-pandemic resilience occurs in the context of trauma exposure and involves lower levels of psychological distress and higher levels of positive psychological well-being. Resilience may lead to higher levels of approach coping and lower levels of avoidant coping in the context of subsequent stressors, measured during the pandemic. Our framework also acknowledges that there may be bidirectional pathways between coping, psychological distress and well-being. For example, higher resilience may predict that individuals would later engage in more adaptive coping; at the same time, adaptive coping could also facilitate resilient outcomes in those individuals in the first place. To examine the potential influence of pre-pandemic resilience on coping during the COVID-19 pandemic, this study drew on data from the Nurses’ Health Study II (NHS II), a large-scale cohort of female registered nurses across the US who have been followed since 1989. Women were registered nurses at the time of the original study recruitment and 26.5% were working as healthcare professionals during the early months of the pandemic. Both trauma exposure and psychological health were measured in this cohort at multiple time points prior to 2020, allowing us to assess pre-pandemic resilience prospectively. Following one established strategy for defining resilience (Masten et al., 2004; Jung et al., 2021; Nishimi et al. 2021), we assessed psychological health relative to trauma burden among those who experienced trauma. We use a residuals-based approach [33], where a continuous measure of resilience was created by regressing continuous psychological health scores on continuous trauma burden and the standardized residuals from this regression model were output. If an individual showed higher psychological health (low distress and high well-being) than would be expected based on their trauma burden they were considered to have a high level of resilience. In contrast, if individuals showed lower psychological health than would be expected based on their trauma burden, they were considered to have lower levels of resilience. Resilience is a complex construct but by accounting for critical elements such as trauma burden, psychological distress and psychological health, we were able to use a single metric to measure it.

NHS II participants also reported information on coping behaviors during the pandemic at one time point between May and August in 2020, after the COVID-19 pandemic began. In this study, we examined resilience to lifetime trauma occurring before the pandemic and its relationship to coping styles and strategies during the COVID-19 pandemic in 2020. We aimed to a) identify types of coping strategies that were frequently used during the pandemic in the context of high or low pre-pandemic resilience quartiles, b) examine pre-pandemic resilience as a potential predictor of coping styles and strategies during 2020, and c) assess potential effect modification by active healthcare professional status. We hypothesized that resilience would be associated with greater use of the approach coping style and strategies, and with decreased use of the avoidant coping style and strategies. We also included an exploratory aim, to consider whether active healthcare professionals might employ different coping styles or strategies compared to counterparts not working as healthcare professionals during the pandemic, given the chronic stress felt by frontline healthcare professionals in the early months of the COVID-19 pandemic (Di Monte et al., 2020).

## Method

### Study sample

The NHS II is a longitudinal cohort study of 116,429 female registered nurses in the US who were aged 25–42 upon enrollment in 1989. Participants complete biennial questionnaires and follow-up is ongoing. The biennial questionnaire from 2017 included psychological health measures and a subset of the cohort (N = 33,845, 65.7% response rate) completed a supplemental posttraumatic stress disorder (PTSD) questionnaire in August 2018- December 2019 which assessed exposure to lifetime trauma and psychological health. Furthermore, in April 2020, active and eligible NHS II participants were invited to complete a baseline survey and a series of monthly follow-up surveys regarding health and well-being during the covid-19 pandemic. A total of 16,717 participants completed both the PTSD and COVID-19 pandemic related surveys. We were interested in examining differences among trauma-exposed individuals, with acknowledgment that by definition, resilience implies the presence of adversity (Choi et al., 2019). Therefore, we restricted the analytic sample to participants who had complete data on all variables needed to create our measure of resilience (2017 measurement of anxiety, 2018 measurement of depression and posttraumatic stress symptoms, 2017 measurement of life satisfaction, optimism and purpose; N = 15,962), who reported at least one lifetime traumatic event prior to the pandemic, and had data on our coping outcome, giving us a final sample size of N = 13,143. A flow chart detailing participant sample derivation is available in S1 Fig. This study was approved by the Partners Healthcare Human Research Committee and participant return of questionnaires implied informed consent, but written or verbal consent was not documented or witnessed. The authors did not have access to information that could identify individual participants during or after data collection.

### Measures

**Fig 2:**
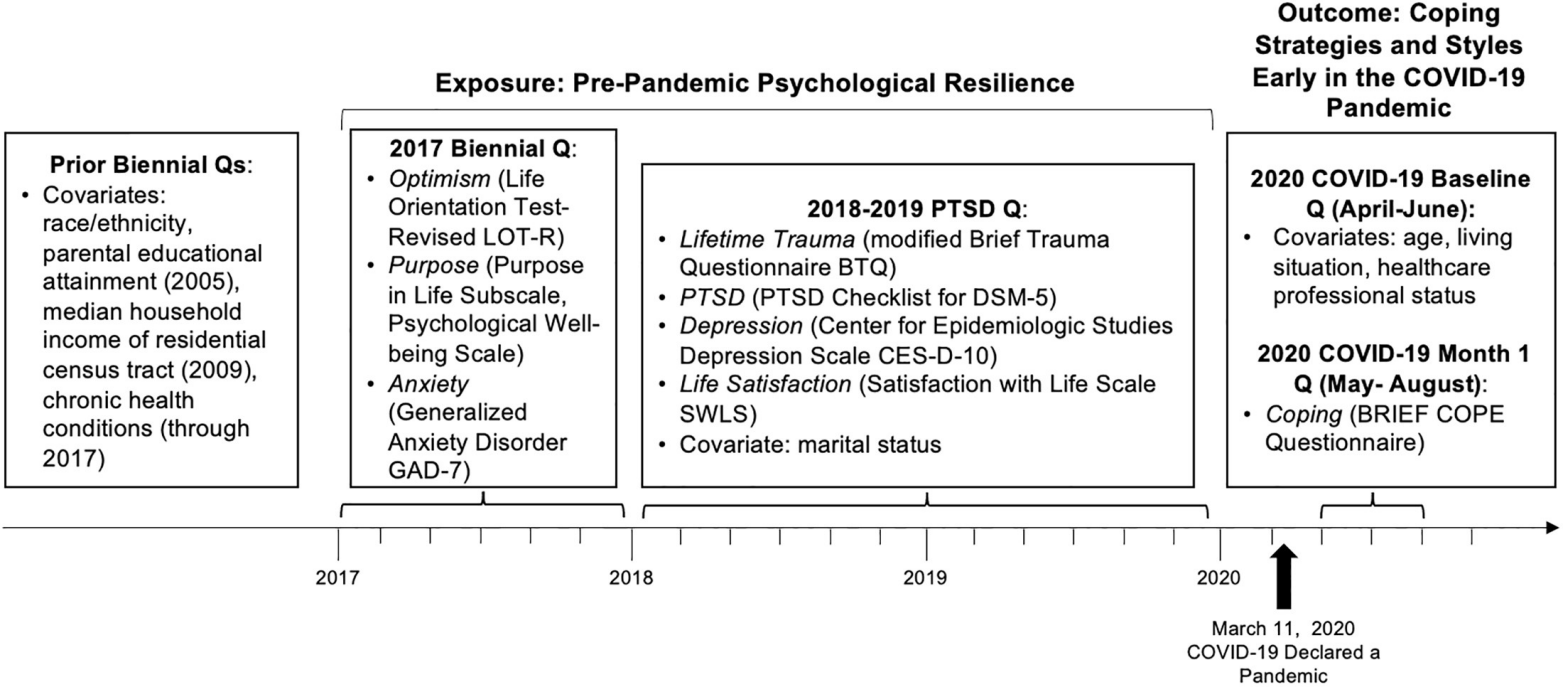
Study measurement timeline.

The timing of specific measures is summarized in Fig 2.

#### Independent variable: Resilience prior to the COVID-19 pandemic

Psychological resilience was operationalized using two constructs, lifetime trauma burden and psychological health, following prior work in this cohort (Choi et al., 2022). Lifetime trauma exposure was reported on the supplemental PTSD questionnaire (August 2018-December 2019) using a modified version of the Brief Trauma Questionnaire [34], which assessed lifetime experience of 16 types of potentially traumatic events, including a write-in category for events not specified. Trauma burden was derived by calculating a count of total trauma types endorsed (potential range 1–16). Please refer to Sampson and colleagues work for a list of all trauma types[35]. This method of measuring trauma burden is consistent with literature suggesting that many individuals experience multiple traumas over their lifetime and trauma has a cumulative negative effect on health [36,37]. Additionally, some research has suggested that different trauma types may not be related to subsequent symptom severity [38]. Following prior work [39], psychological health was defined by combining measures of distress and positive psychological well-being to capture the mental health spectrum (Winefield et al., 2012; Nishimi et al., 2021). Distress was measured using self-reported past month posttraumatic stress symptoms with the 20-item PTSD Checklist for the DSM-5 [40] (PCL-5); depressive symptoms were measured with the 10-item Center for Epidemiologic Studies Depression Scale [41] (CES-D); and anxiety symptoms were measured with the 7 item GAD-7 scale [42]. Both PTSD and depression measures were included on the supplemental PTSD questionnaire (August 2018- December 2019); anxiety was included in 2017 biennial questionnaire. Positive psychological well-being was measured by combining measures of life satisfaction [43] taken from the supplemental PTSD questionnaire, optimism (6-item Life Orientation Test-Revised [44]) and purpose (3-item purpose in life subscale of the Psychological Well-being Scale [45] from the 2017 biennial questionnaire. Sum scores were created for each separate distress and positive psychological well-being scale and each overall score was then standardized (M = 0, SD = 1). Next, all scores were summed to create a composite psychological health score, inverting the distress scores beforehand such that higher total sum scores indicate more positive psychological health. This method of creating a composite score which included elements of distress and positive well-being is consistent with other work [28,46] and reflects a continuum of distress and positive functioning to define psychological health broadly, rather than focusing solely on the absence of distress which may not necessarily be indicative of healthy functioning [47]. Psychological well-being is complex and multidimensional [48], therefore including different dimensions to characterize a range of potential psychological responses to trauma was necessary. A confirmatory factor analysis suggested that measures of distress and positive psychological well-being load acceptably on a single factor (SRMR = 0.058; BCFI = 0.89).

Although not all measures of psychological health were available at all time points, these forms of distress and positive well-being have been shown to be stable during adulthood [49–51], suggesting it is reasonable to combine measures from the 2017 and 2018–2019 surveys. Assessment of lifetime trauma was taken after some of the psychological health measurements, but all “worst” trauma events (on measurements of posttraumatic stress, participants are asked to indicate which trauma they consider the “worst” they had experienced and respond to queries about trauma-related symptoms with that event in mind) were reported as occurring before 2017, with a mean age of occurrence as 34 years (SD = 17).

A residual-based approach was used to create a continuous measure of resilience [33] by regressing the psychological health score on the count measure of trauma burden. Standardized residuals derived from this regression model reflect the difference between actual and expected psychological health. If a participant’s psychological health score was higher than predicted by the regression with trauma burden, the resulting residual indicates higher levels of resilience; if the score was lower than predicted, the resulting residual indicates lower levels of resilience.

#### Dependent outcome variable: Coping during the COVID-19 pandemic

Coping was measured using the BRIEF COPE questionnaire [52] which includes 15 items rated on a Likert scale of 1 “I haven’t been doing this at all” to 4 “I’ve been doing this a lot”. Each query is about current coping behaviors, in the past seven days measured at the first follow-up questionnaire following baseline (called Month 1, collected between June and September 2020) of the COVID-19 questionnaire. The 15 items comprise nine coping strategy domains (self-distraction, behavioral disengagement, self-blame, substance use, emotional support, positive reframing, active coping [indicating the use of one’s own resources to problem-solve], humor, and religion) each with a mean response score ranging between 1 and 4 [53]. Some items were adapted to clarify “it” as “the stress I’m experiencing” but items were not adapted specifically to bring attention to the pandemic. For example, the substance use coping strategy item read, “In the past 7 days, I’ve been using alcohol or other drugs to help me get through the stress I’m experiencing.” Two coping style subscales (approach coping and avoidant coping) [10] can be created by calculating a mean score for items contributing to each subscale ranging between 1 and 4. The approach coping style subscale contains items measuring active coping, emotional support, and positive reframing strategies. The avoidant coping style subscale includes items measuring self-distraction, behavioral disengagement, self-blame, and substance use strategies. Cronbach’s alpha for internal consistency was good for both the approach coping subscale (α = 0.88) and the avoidant coping subscale (α = 0.72).

#### Covariates

Covariates included age (in years in 2020); race/ethnicity (white or non-white) reported on the 1989 NHS II biennial questionnaire; marital status (married, divorced/separated, widowed, single, other/missing) reported on the supplemental PTSD questionnaire in 2018–2019; presence of chronic health conditions (history of any cancer, stroke, or heart attack) reported on biennial questionnaires through 2017; living situation (with others versus alone) reported on the COVID-19 baseline questionnaire; and active healthcare professional status (current healthcare professional, not a current healthcare professional) reported on the COVID-19 baseline questionnaire. We also included parental education attainment (highest level of education completed by either parent: high school graduate, 1–3 years of college, or 4 years of college or greater, missing) reported on the 2005 biennial questionnaire, as an indicator of childhood socioeconomic status and median household income of residential census tract (in quartiles) for residential locations in 2009, as an indicator of adult socioeconomic status. Both represent potential confounders as socioeconomic status in childhood or adulthood could be related to lifetime trauma, psychological health, and coping.

### Statistical analyses

For descriptive purposes, we first examined the distribution of resilience and coping strategies among our analytic sample and compared the mean scores on each coping strategy domain between those scoring in the highest and lowest resilience quartiles. We examined the frequency of use of individual coping strategies and correlations among resilience, coping styles and coping strategies. We also examined unadjusted bivariate regressions between pre-pandemic resilience and coping styles and strategies.

Second, for our primary analyses we used multiple linear regression models to examine associations between continuous pre-pandemic resilience scores and higher use of approach or avoidant coping styles during the pandemic, adjusting for all covariates. Next, we used multiple linear regression models to examine associations between pre-pandemic resilience and use of each of the nine coping strategies (distraction, behavioral disengagement, self-blame, substance use, emotional support, positive reframing, active coping, humor, and religion) separately, adjusting for all covariates. We did this to assess if pre-pandemic resilience was related to specific coping strategies and to examine whether certain strategies were driving the associations between resilience and approach or avoidant styles. For interpretability, we calculated standardized beta estimates by standardizing all continuous variables (i.e., predictors and outcomes).

#### Sensitivity analyses

As healthcare professionals may experience higher levels of stress during the pandemic than non-healthcare professionals, we examined whether effects of pre-pandemic resilience on coping might differ depending on healthcare professional status, by including an appropriate interaction term in each linear regression model (pre-pandemic continuous resilience for each coping outcome separately).

## Results

Table 1 summarizes the distribution of covariates by mean resilience score among the full trauma-exposed sample. Women in this sample were on average 67 years old (SD = 4.5), primarily white (96.1%), and married or partnered (75.2%). A total of 26.5% of our sample were active healthcare professionals and 14.8% were living with a chronic health condition. Educational attainment by the parents of participants was varied, with 46.4% having completed high school as their highest education level. While all women in the analytic sample had experienced at least one lifetime trauma prior to the pandemic, 50% reported experiencing 3 or more types of trauma, denoting a high trauma burden. Each additional trauma type experienced was associated with -0.53 standard deviation reduction in psychological health prior to the pandemic.

**Table 1:** Distribution of model covariates in the analytic sample and mean resilience scores by covariates in NHS2 COVID-19 substudy participants (N = 13,143).

Covariate	n (%)	Mean Resilience Score (SD)	p-value (overall)
Race			.25
White	12628 (96.1)	-.00 (1.0)	
Non-white	377 (2.9)	.08 (.92)	
Parent Education			**.001**
High School	6104 (46.4)	-.03 (1.0)	
Some College	3090 (23.5)	.02 (.98)	
College Plus	3287 (25.0)	.04 (.98)	
Median Income 2009			**< .001**
Quartile 1 (25%)	3287 (25.0)	-.04 (1.0)	
Quartile 2 (50%)	3274 (24.9)	-.03 (1.0)	
Quartile 3 (75%)	3293 (25.1)	.01 (.97)	
Quartile 4 (100%)	3267 (24.9)	.06 (.95)	
Marital Status			**< .0001**
Married/ Partnered	9886 (75.2)	.07 (.94)	
Divorced/ Separated	1645 (12.5)	-.23 (1.2)	
Widowed	863 (6.6)	-.17 (1.1)	
Single	669 (5.1)	-.31 (1.1)	
Current Living Arrangement			**< .0001**
With Others	10633 (80.9)	.06 (.95)	
Alone	2112 (16.1)	-.24 (1.1)	
Active Healthcare Professional			.08
Yes	3479 (26.5)	.03 (.96)	
No	9662 (73.5)	-.01 (1.0)	
Living with Chronic Health Condition			.98
Yes	1940 (14.8)	.00 (1.0)	
No	11203 (85.2)	-.00 (.99)	

Note: p < .05 are **bolded**; p-values refer to T-tests for binary categorical covariates and F-statistics for categorical covariates (>2 categories).

### Descriptive analyses

**Fig 3:**
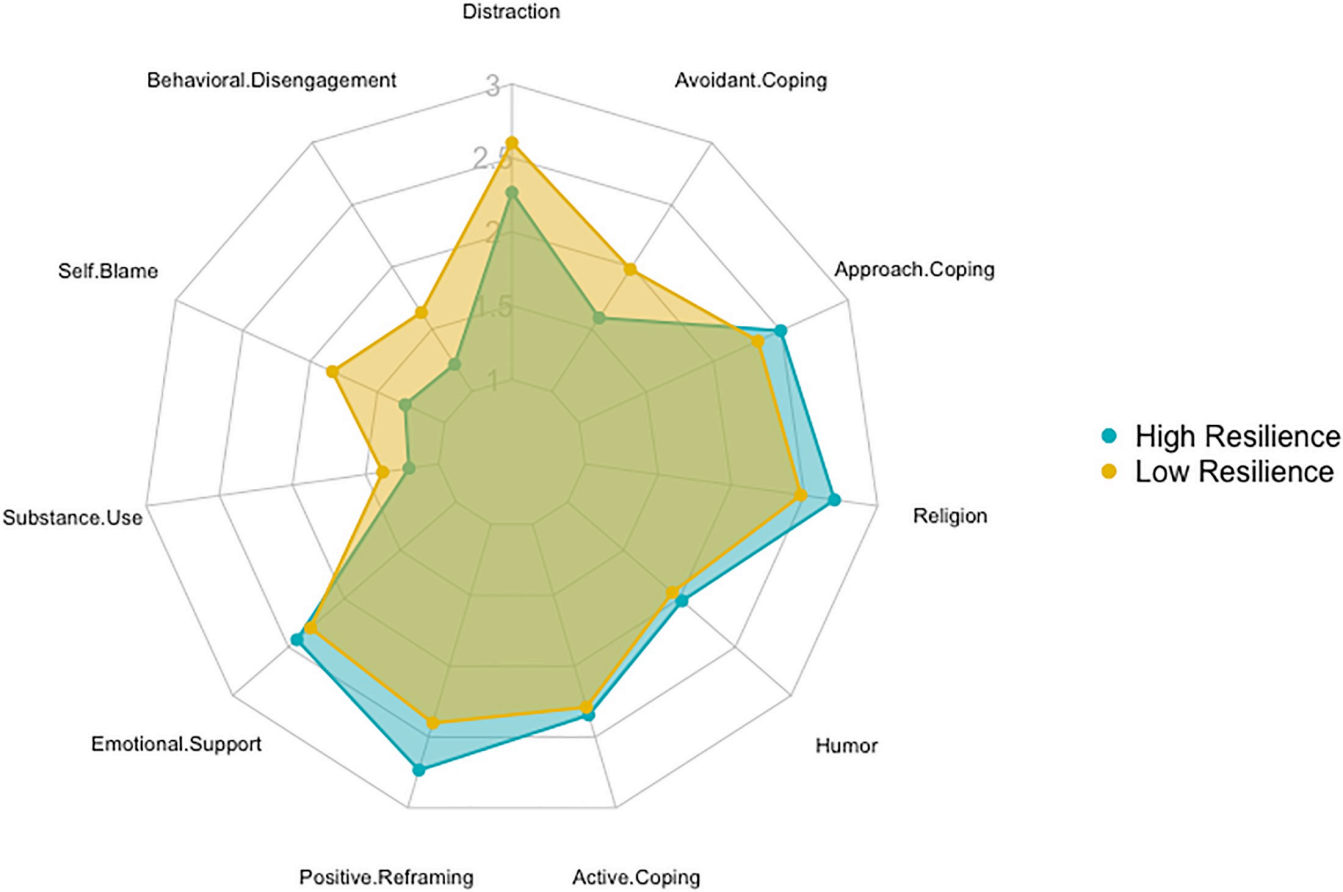
Mean coping strategy use by highest and lowest resilience quartile.

To examine coping strategies across women with higher versus lower resilience, Fig 3 presents the mean frequency of use of each of the nine coping strategies by participants in the highest versus lowest quartile of the continuous resilience score. We observed patterns of coping strategies, with participants with the highest pre-pandemic resilience endorsing slightly greater use of emotional support, religion, and positive reframing coping strategies during the pandemic than their lowest resilience counterparts. Participants with the lowest levels of pre-pandemic resilience used distraction, self-blame, behavioral disengagement, and substance use coping strategies during the pandemic slightly more frequently than their high resilience counterparts. However, substance use, self-blame and behavioral disengagement were the least frequently utilized strategies overall. Positive reframing, religion and distraction were the most used strategies overall.

In S1 Table, we present Pearson correlation coefficients between resilience, coping styles and individual coping strategies. Correlations between the individual coping strategies were small to moderate.

### Pre-pandemic psychological resilience and coping style during the pandemic

In our primary multiple linear regression models, continuous pre-pandemic resilience was significantly associated with both avoidant and approach coping styles (separately) during the pandemic in expected directions. Specifically, greater pre-pandemic resilience was associated with lower use of an avoidant coping style (ß = -0.39, 95% CI -0.41, -0.38) and higher use of an approach coping style (ß = 0.06, 95% CI 0.05, 0.08) during the pandemic in linear regression after adjusting for age, race, parental educational attainment, healthcare professional status, chronic health condition status and income (see Table 2; see S2 Table for covariate estimates).

**Table 2:** Linear regression model estimate: Pre-pandemic resilience predicting coping styles and strategies during the pandemic.

	Pre-pandemic Resilience to Trauma			
	ß	95% CI	*p*	ƒ^2^
Coping Style				
Approach Coping	.06	.05,.08	<0.0001	0.01
Avoidant Coping	-.39	-.41, -.38	<0.0001	0.16
Coping Strategies				
Distraction	-.18	-.20, -.16	<0.0001	0.04
Substance Use	-.15	-.17, -.13	<0.0001	0.03
Behavioral Disengagement	-.29	-.30, -.27	<0.0001	0.08
Self-Blame	-.44	-.45, -.42	<0.0001	0.19
Emotional Support	.03	.01.05	<0.0001	0.01
Positive Reframing	.13	.12,.15	<0.0001	0.03
Active Coping	-.00	-.02,.01	0.7852	0.01
Humor	.03	.01,.05	<0.0001	0.01
Religion	.06	.04,.08	<0.0001	0.03

Note: All models adjusted for age, race, parental educational attainment, census tract-level median household income, marital status, current living arrangement, healthcare professional status, and chronic condition status.

Age, coping, and resilience variables were standardized for interpretability.

### Pre-pandemic psychological resilience and use of coping strategies during the pandemic

In a series of multiple linear regression models, continuous pre-pandemic resilience was significantly associated with use of eight of the nine coping strategies assessed during the pandemic in the expected directions, but the magnitude of the associations varied substantially. Pre-pandemic resilience was negatively associated with use of individual avoidant strategies (e.g., distraction [ß = -0.18, 95% CI -0.20, -0.16], substance use [ß = -0.15, 95% CI -0.17, -0.13], behavioral disengagement [ß = -0.29, 95% CI -0.30, -0.27], self-blame [ß = -0.44, 95% CI -0.45, -0.42]). Pre-pandemic resilience was positively associated with use of two individual approach strategies: emotional support (ß = 0.03, 95% CI 0.01, 0.05) and positive reframing (ß = 0.13, 95% CI 0.12, 0.15). Pre-pandemic resilience was also positively associated with use of humor (ß = 0.03, 95% CI 0.01, 0.05) and religion (ß = 0.06, 95% CI 0.04, 0.08). It was not significantly associated with the active coping strategy (ß = -0.00, 95% CI -0.02, 0.01). All models adjusted for age, race, parental educational attainment, healthcare professional status, chronic health condition status and income. Effect sizes were small, defined as between 0.02–0.14 except for the self-blame coping strategy which had a medium size (defined as between 0.15–0.35; [54], suggesting that there was a statistically significant effect but that the magnitude of the association was small to medium. A summary of these results is available in Table 2. Unadjusted associations did not differ in terms of statistical significance. These associations are available in S3 Table.

### Sensitivity analyses

We found no evidence of interaction between resilience and current healthcare professional status, except for the avoidant coping style (β = 0.03, p = 0.04). Models were not further stratified by healthcare professional status.

## Discussion

Our findings provide insights into how women with prior histories of trauma were coping during the early months of a global pandemic. Women in our sample, both active and former healthcare professionals, used a variety of strategies to cope with life stress during the pandemic. Active healthcare professionals did not appear to use significantly different coping strategies or styles from those not currently working in the healthcare field. Whether they scored high or low on pre-pandemic resilience to trauma, there was substantial overlap in the frequency of specific coping strategies utilized during the pandemic. However, overall, pre-pandemic resilience was significantly associated with greater use of an approach coping style during the pandemic and particularly with lower use of an avoidant coping style. Given prior work showing approach coping style as more adaptive compared to an avoidant coping style, resilience may promote better health outcomes through its association with adaptive coping during times of stress [6,11]. This finding highlights the impact of previous resilience to traumatic experiences on one’s ability to engage with more adaptive coping strategies during new times of stress.

Our findings suggest that women used numerous individual coping strategies during the pandemic but also that those with higher pre-pandemic resilience to trauma were significantly more likely to use certain strategies. In our sample, strategies such as seeking emotional support, positive reframing, and religion were more frequently reported by individuals in the highest resilience group and were positively associated with resilience. Frequency of use of humor and active coping strategies did not differ according to levels of pre-pandemic psychological resilience; they were used approximately equivalently by those in the highest and lowest resilience groups. However, while humor was positively associated with resilience, perhaps surprisingly, active coping was not significantly related to levels of pre-pandemic psychological resilience. This null finding suggests that resilience may not associate with this particular coping strategy. Active coping is often seen as adaptive and how effective the strategy is or how utilized it is may be context dependent [7], and perhaps in the context of the COVID-19 pandemic, active coping was less frequently utilized or experienced as helpful. On the other hand, specific coping strategies such as substance use, self-blame, and behavioral disengagement, which were infrequently used overall during the pandemic, were used significantly more frequently by individuals with lower resilience scores. Distraction was frequently endorsed during the pandemic overall in our sample but was also negatively related to pre-pandemic resilience. Although distraction is generally understood as a more maladaptive coping strategy, it might be expected that in the early months of the COVID-19 pandemic many women might engage in distraction to cope as they were bombarded with constant stressful information related to COVID-19. Generally, there was a stronger association between pre-pandemic resilience and lower use of avoidant strategies, than there was between pre-pandemic resilience and higher use of approach strategies. This finding suggests resilience may influence women to engage in less maladaptive behaviors during times of stress. Also, it may imply that more resilient women were engaging in a greater variety of strategies to cope. This would be consistent with prior literature suggesting that moderate variability in coping strategies is more adaptive then employing only one or two strategies repeatedly in the face of different stressors [9].

Coping strategies used during the pandemic that were linked to higher pre-pandemic resilience may be similar to strategies more resilient women used previously to cope effectively with other forms of trauma; thus, pre-pandemic resilience may primarily serve as a marker of adaptive coping tendencies. Alternatively, pre-pandemic resilience may promote future adaptive coping, as experiencing greater psychological health despite prior trauma may provide a foundation allowing trauma-exposed women to engage more effectively in adaptive coping strategies in times of future stress. These pathways would be consistent with the PASTOR theory [25] and integrative affect-regulation framework [26] which informed the design of the current work.

Our study can inform future longitudinal work examining earlier resilience to trauma and coping experiences during other types of collectively stressful times. In addition, the long-term mental health impacts of specific coping strategies used during the pandemic should be explored. Examination of whether coping strategies or styles mediate effects of higher psychological resilience with regard to subsequent mental health outcomes is an important next step. Such work could lead to guidance in clinical practice, to assess coping styles and teach adaptive coping strategies to contribute to future psychological health. In addition, a purposefully built comprehensive measure of resilience in a prospective dataset could further expand our understanding of the construct.

The present study has several strengths, including a prospective design in a large, well-established cohort of women. We were able to explore how vulnerable individuals may cope during the COVID-9 pandemic and utilized a nuanced definition of resilience which incorporated prior trauma exposure and psychological health into a single predictive measure.

Our study also has limitations. Our findings may not generalize as our sample primarily consisted of older white female healthcare professionals. Future research should examine these questions in more vulnerable populations, including low-income and minoritized individuals, who may experience greater levels of lifetime trauma. Some research has suggested that cultural factors influence the development of resilience [55] and replication in more diverse samples could better explore this possibility. However, even in a relatively advantaged and educated sample, lower pre-pandemic resilience was still associated with an avoidant coping style and coping strategies that are widely viewed as more maladaptive (e.g., substance use). We also adjusted for several confounders that could influence coping, but residual confounding or unmeasured confounding remains a possibility. Coping was assessed at a single timepoint, providing a snapshot of how women coped with stress early in the pandemic. A separate question relates to whether and how resilience might affect changes in coping strategy use over time; it is possible that resilience reliably promotes more adaptive coping during different periods of stress. However, due to data availability and scope, the current study did not explore the inter-relationships between resilience and use of coping strategies over time. Moreover, although our resilience measures preceded coping measures, it is possible that more adaptive coping initially led women to have higher pre-pandemic resilience and more adaptive coping as well during the pandemic. This potential bidirectional relationship warrants further attention. Nonetheless, our results suggest that manifested resilience at one time point can serve as a prospective marker of coping behavior during future stressful experiences. While the present study examined general coping behaviors during the pandemic, future work could also examine coping behaviors that were specific to managing the COVID-19 pandemic. Future research may also want to examine whether approach coping related to prior resilience translates into better health outcomes. Examining the co-occurrence of specific coping strategies, how they cluster and how flexible one’s coping styles are would push the work in this area forward, contextualizing our findings and those of others [9,56]. The relationship between psychological health and coping is likely bidirectional and as our conceptualization of resilience involves measures of psychological health, we are only able to examine the relationship in a single direction. Finally, trauma burden was assessed as a count of the number of traumatic event types, as our focus was more on the granularity of coping behavior outcomes rather than types of trauma experienced. Future research should delve into the complexity or severity of specific traumatic events. Future research should also seek to further hone a comprehensive operationalization of resilience, that is relevant to groups at particular risk for exposure to adversity.

In conclusion, by examining longitudinal data from over 13,000 women with detailed psychosocial information both before and during the COVID-19 pandemic, we found that higher resilience to prior trauma was associated with more adaptive coping styles and greater use of several specific coping strategies—those characterized primarily by approach rather than avoidance, during the pandemic. Our findings have important implications for intervention or even prevention efforts to support vulnerable groups, such as women with prior trauma histories, during this and other immensely stressful times. Coping strategies can be taught and identification of specific coping strategies associated with adaptive mental health outcomes offers insight into how women with trauma histories can effectively cope with the many stresses of the COVID-19 pandemic, and potentially future stresses as well. Building psychological resilience following exposure to trauma through intervention and support may provide the basis for effective coping in times of future stress.

## Supporting information

S1 Checklist STROBE statement—Checklist of items that should be included in reports of observational studies. (DOCX)

S1 Fig Participant derivation summary. (TIFF)

S1 Table Pearson’s correlation coefficients among resilience, coping styles and coping strategies. (PDF)

S2 Table Linear regression models: Covariates predicting coping styles and strategies. (PDF)

S3 Table Unadjusted associations between resilience and coping styles and strategies. (PDF)

## References

[1] Montero-Marin J, Prado-Abril J, Piva Demarzo MM, Gascon S, García-Campayo J. Coping with stress and types of burnout: explanatory power of different coping strategies. PloS one. 2014;9(2):e89090–e. doi: 10.1371/journal.pone.0089090 24551223 PMC3923838

[2] Zheng J, Morstead T, Sin N, Klaiber P, Umberson D, Kamble S, et al. Psychological distress in North America during COVID-19: The role of pandemic-related stressors. Social science & medicine (1982). 2021;270:113687-. doi: 10.1016/j.socscimed.2021.113687 33465600 PMC9757831

[3] Benight CC, Harper ML. Coping self-efficacy perceptions as a mediator between acute stress response and long-term distress following natural disasters. Journal of Traumatic Stress: Official Publication of The International Society for Traumatic Stress Studies. 2002;15(3):177–86. doi: 10.1023/A:1015295025950 12092909

[4] Cheng C, Cheung MWL. Psychological Responses to Outbreak of Severe Acute Respiratory Syndrome: A Prospective, Multiple Time-Point Study. Journal of personality. 2005;73(1):261–85. doi: 10.1111/j.1467-6494.2004.00310.x 15660679 PMC7167099

[5] Main A, Zhou Q, Ma Y, Luecken LJ, Liu X. Relations of SARS-Related Stressors and Coping to Chinese College Students’ Psychological Adjustment During the 2003 Beijing SARS Epidemic. Journal of counseling psychology. 2011;58(3):410–23. doi: 10.1037/a0023632 21574694

[6] Kar N, Kar B, Kar S. Stress and coping during COVID-19 pandemic: Result of an online survey. Psychiatry research. 2021;295:113598-. doi: 10.1016/j.psychres.2020.113598 33264677 PMC7688436

[7] Shamblaw AL, Rumas RL, Best MW. Coping During the COVID-19 Pandemic: Relations With Mental Health and Quality of Life. Canadian psychology = Psychologie canadienne. 2021;62(1):92–100. doi: 10.1037/cap0000263

[8] Stanistawski K. The Coping Circumplex Model: An Integrative Model of the Structure of Coping With Stress. Frontiers in psychology. 2019;10:694-. doi: 10.3389/fpsyg.2019.00694 31040802 PMC6476932

[9] Cheng C, Lau H-PB, Chan M-PS. Coping flexibility and psychological adjustment to stressful life changes: a meta-analytic review. Psychological bulletin. 2014;140(6):1582. 25222637 10.1037/a0037913

[10] Eisenberg SA, Shen B-J, Schwarz ER, Mallon S. Avoidant coping moderates the association between anxiety and patient-rated physical functioning in heart failure patients. Journal of behavioral medicine. 2011;35(3):253–61. doi: 10.1007/s10865-011-9358-0 21660588

[11] Gurvich C, Thomas N, Thomas EHX, Hudaib A-R, Sood L, Fabiatos K, et al. Coping styles and mental health in response to societal changes during the COVID-19 pandemic. International journal of social psychiatry. 2021;67(5):540–9. doi: 10.1177/0020764020961790 33016171

[12] Krause ED, Kaltman S, Goodman LA, Dutton MA. Avoidant coping and PTSD symptoms related to domestic violence exposure: A longitudinal study. Journal of traumatic stress. 2008;21(1):83–90. doi: 10.1002/jts.20288 18302182

[13] Leiner AS, Kearns MC, Jackson JL, Astin MC, Rothbaum BO. Avoidant Coping and Treatment Outcome in Rape-Related Posttraumatic Stress Disorder. Journal of consulting and clinical psychology. 2012;80(2):317–21. doi: 10.1037/a0026814 22229757 PMC3314118

[14] Street AE, Gibson LE, Holohan DR. Impact of childhood traumatic events, trauma-related guilt, and avoidant coping strategies on PTSD symptoms in female survivors of domestic violence. Journal of traumatic stress. 2005;18(3):245–52. doi: 10.1002/jts.20026 16281219

[15] Park CL, Finkelstein-Fox L, Russell BS, Fendrich M, Hutchison M, Becker J. Psychological Resilience Early in the COVID-19 Pandemic: Stressors, Resources, and Coping Strategies in a National Sample of Americans. The American psychologist. 2021;76(5):715–28. doi: 10.1037/amp0000813 34081505 PMC8595499

[16] Khalaf OO, Khalil MA, Abdelmaksoud R. Coping with depression and anxiety in Egyptian physicians during COVID-19 pandemic. Middle East current psychiatry (Cairo). 2020;27(1):1–7. doi: 10.1186/s43045-020-00070-9

[17] Mi T, Yang X, Sun S, Li X, Tam CC, Zhou Y, et al. Mental Health Problems of HIV Healthcare Providers During the COVID-19 Pandemic: The Interactive Effects of Stressors and Coping. AIDS and behavior. 2020;25(1):18–27. doi: 10.1007/s10461-020-03073-z 33128108 PMC7598225

[18] Di Monte C, Monaco S, Mariani R, Di Trani M. From Resilience to Burnout: Psychological Features of Italian General Practitioners During COVID-19 Emergency. Frontiers in psychology. 2020;11:567201-. doi: 10.3389/fpsyg.2020.567201 33132972 PMC7566043

[19] Luthar SS, Cicchetti D, Becker B. The Construct of Resilience: A Critical Evaluation and Guidelines for Future Work. Child development. 2000;71(3):543–62. doi: 10.1111/1467-8624.00164 10953923 PMC1885202

[20] Masten AS, Best KM, Garmezy N. Resilience and development: Contributions from the study of children who overcome adversity. Development and psychopathology. 1990;2(4):425–44. doi: 10.1017/S0954579400005812

[21] Rutter M. Psychosocial resilience and protective mechanisms. Cambridge University Press; 1990. p. 181–214.

[22] Bonanno GA. Loss, Trauma, and Human Resilience: Have We Underestimated the Human Capacity to Thrive After Extremely Aversive Events? The American psychologist. 2004;59(1):20–8. doi: 10.1037/0003-066X.59.1.20 14736317

[23] Connor KM, Davidson JRT. Development of a new resilience scale: The Connor-Davidson Resilience Scale (CD-RISC). Depression and anxiety. 2003;18(2):76–82. doi: 10.1002/da.10113 12964174

[24] Choi KW, Stein MB, Dunn EC, Koenen KC, Smoller JW. Genomics and psychological resilience: a research agenda. Molecular psychiatry. 2019;24(12):1770–8. doi: 10.1038/s41380-019-0457-6 31341239 PMC6874722

[25] Kalisch R, Müller MB, Tüscher O. A conceptual framework for the neurobiological study of resilience. The Behavioral and brain sciences. 2015;38:e92–e. doi: 10.1017/S0140525X1400082X 25158686

[26] Troy AS, Willroth EC, Shallcross AJ, Giuliani NR, Gross JJ, Mauss IB. Psychological Resilience: An Affect-Regulation Framework. Annual review of psychology. 2023;74(1):547–76. doi: 10.1146/annurev-psych-020122-041854 36103999 PMC12009612

[27] Koenen KC. Developmental Epidemiology of PTSD: Self-Regulation as a Central Mechanism. Annals of the New York Academy of Sciences. 2006;1071(1):255–66. doi: 10.1196/annals.1364.020 16891576

[28] Winefield HR, Gill TK, Taylor AW, Pilkington RM. Psychological well-being and psychological distress: is it necessary to measure both? Psychology of well-being. 2012;2(1):3. doi: 10.1186/2211-1522-2-3

[29] Choi KW, Nishimi K, Jha SC, Sampson L, Hahn J, Kang JH, et al. Pre-pandemic resilience to trauma and mental health outcomes during COVID-19. Social Psychiatry and Psychiatric Epidemiology. 2022:1–13. doi: 10.1007/s00127-022-02367-y 36169684 PMC9514982

[30] Bandoli G, Campbell-Sills L, Kessler RC, Heeringa SG, Nock MK, Rosellini AJ, et al. Childhood adversity, adult stress, and the risk of major depression or generalized anxiety disorder in US soldiers: a test of the stress sensitization hypothesis. Psychological medicine. 2017;47(13):2379–92. doi: 10.1017/S0033291717001064 28443533 PMC5595661

[31] McLaughlin KA, Conron KJ, Koenen KC, Gilman SE. Childhood adversity, adult stressful life events, and risk of past-year psychiatric disorder: a test of the stress sensitization hypothesis in a population-based sample of adults. Psychological medicine. 2010;40(10):1647–58. doi: 10.1017/S0033291709992121 20018126 PMC2891275

[32] Snijders C, Pries L-K, Sgammeglia N, Jowf GA, Youssef NA, De Nijs L, et al. Resilience Against Traumatic Stress: Current Developments and Future Directions. Frontiers in psychiatry. 2018. doi: 10.3389/fpsyt.2018.00676 30631285 PMC6315131

[33] Amstadter AB, Maes HH, Sheerin CM, Myers JM, Kendler KS. The relationship between genetic and environmental influences on resilience and on common internalizing and externalizing psychiatric disorders. Social Psychiatry and Psychiatric Epidemiology. 2015;51(5):669–78. doi: 10.1007/s00127-015-1163-6 26687369 PMC5137200

[34] Schnurr P, Vielhauer M, Weathers F, Findler M. The brief trauma questionnaire (BTQ)[measurement instrument]. US Department of Veterans Affairs. 1999.

[35] Sampson L, Jha SC, Roberts AL, Lawn RB, Nishimi KM, Ratanatharathorn A, et al. Trauma, Post-Traumatic Stress Disorder, and Treatment Among Middle-Aged and Older Women in the Nurses’ Health Study II. The American journal of geriatric psychiatry. 2022;30(5):588–602. doi: 10.1016/j.jagp.2021.10.017 34916131 PMC8983445

[36] Chartier MJ, Walker JR, Naimark B. Separate and cumulative effects of adverse childhood experiences in predicting adult health and health care utilization. Child abuse & neglect. 2010;34(6):454–64. doi: 10.1016/j.chiabu.2009.09.020 20409586

[37] Hamby S, Elm JHL, Howell KH, Merrick MT. Recognizing the cumulative burden of childhood adversities transforms science and practice for trauma and resilience. The American psychologist. 2021;76(2):230–42. doi: 10.1037/amp0000763 33734791

[38] Ruork AK, McLean CL, Fruzzetti AE. It Happened Matters More Than What Happened: Associations Between Intimate Partner Violence Abuse Type, Emotion Regulation, and Post-Traumatic Stress Symptoms. Violence Against Women. 2022;28(5):1158–70. doi: 10.1177/10778012211013895 . 34057860

[39] Nishimi KM, Koenen KC, Coull BA, Chen R, Kubzansky LD. Psychological resilience predicting cardiometabolic conditions in adulthood in the Midlife in the United States Study. Proceedings of the National Academy of Sciences. 2021;118(32). doi: 10.1073/pnas.2102619118 34341103 PMC8364125

[40] Weathers FW, Bovin MJ, Lee DJ, Sloan DM, Schnurr PP, Kaloupek DG, et al. The Clinician-Administered PTSD Scale for DSM-5 (CAPS-5): Development and Initial Psychometric Evaluation in Military Veterans. Psychological assessment. 2018;30(3):383–95. doi: 10.1037/pas0000486 28493729 PMC5805662

[41] Andresen EM, Malmgren JA, Carter WB, Patrick DL. Screening for Depression in Well Older Adults: Evaluation of a Short Form of the CES-D. American journal of preventive medicine. 1994;10(2):77–84. doi: 10.1016/S0749-3797(18)30622-6 8037935

[42] Spitzer RL, Kroenke K, Williams JB, Löwe B. A brief measure for assessing generalized anxiety disorder: the GAD-7. Archives of internal medicine. 2006;166(10):1092–7. doi: 10.1001/archinte.166.10.1092 16717171

[43] Diener E, Emmons RA, Larsen RJ, Griffin S. The Satisfaction With Life Scale. Journal of personality assessment. 1985;49(1):71–5. doi: 10.1207/s15327752jpa4901_13 16367493

[44] Scheier MF, Carver CS, Bridges MW. Distinguishing Optimism From Neuroticism (and Trait Anxiety, Self-Mastery, and Self-Esteem): A Reevaluation of the Life Orientation Test. Journal of personality and social psychology. 1994;67(6):1063–78. doi: 10.1037//0022-3514.67.6.1063 7815302

[45] Ryff CD, Keyes CLM. The structure of psychological well-being revisited. Journal of personality and social psychology. 1995;69(4):719–27. doi: 10.1037//0022-3514.69.4.719 7473027

[46] Pimple P, Lima BB, Hammadah M, Wilmot K, Ramadan R, Levantsevych O, et al. Psychological Distress and Subsequent Cardiovascular Events in Individuals With Coronary Artery Disease. Journal of the American Heart Association. 2019;8(9):e011866–e. doi: 10.1161/JAHA.118.011866 31055991 PMC6512132

[47] Kubzansky LD, Boehm JK, Segerstrom SC. Positive Psychological Functioning and the Biology of Health. Social and personality psychology compass. 2015;9(12):645–60. Epub Kubzansky, L. D., Boehm, J. K., and Segerstrom, S. C. (2015) Positive Psychological Functioning and the Biology of Health. Social and Personality Psychology Compass, 9: 645–660. doi: 10.1111/spc3.12224

[48] Kim ES, Moskowitz JT, Kubzansky LD. Introduction to Special Issue: Interventions to Modify Psychological Well-Being and Population Health. Affective science. 2023;4(1):1–9. doi: 10.1007/s42761-023-00184-3 37064817 PMC10043542

[49] Carver CS, Scheier MF. Dispositional optimism. Trends in cognitive sciences. 2014;18(6):293–9. doi: 10.1016/j.tics.2014.02.003 24630971 PMC4061570

[50] Cheung F, Lucas RE. Assessing the validity of single-item life satisfaction measures: results from three large samples. Quality of life research. 2014;23(10):2809–18. doi: 10.1007/s11136-014-0726-4 24890827 PMC4221492

[51] Salsman JM, Lai J-S, Hendrie HC, Butt Z, Zill N, Pilkonis PA, et al. Assessing psychological well-being: self-report instruments for the NIH Toolbox. Quality of life research. 2014;23(1):205–15. doi: 10.1007/s11136-013-0452-3 23771709 PMC3883907

[52] Carver CS. You want to measure coping but your protocol’s too long: Consider the brief COPE. International journal of behavioral medicine. 1997;4(1):92–100. doi: 10.1207/s15327558ijbm0401_6 16250744

[53] Zeidner M, Endler NS. Handbook of coping: theory, research, applications. New York: Wiley; 1996.

[54] Cohen J. Statistical Power Analysis For The Behavioral Sciences. Hillsdale, NJ: Erlbaum; 1988. 1007- p.

[55] Clauss-Ehlers CS. Sociocultural factors, resilience, and coping: Support for a culturally sensitive measure of resilience. Journal of Applied Developmental Psychology. 2008;29(3):197–212. doi: 10.1016/j.appdev.2008.02.004

[56] Trudel-Fitzgerald C, Chen R, Lee LO, Kubzansky LD. Are coping strategies and variability in their use associated with lifespan? Journal of psychosomatic research. 2022;162:111035-. doi: 10.1016/j.jpsychores.2022.111035 36152346 PMC10410682

