# Supplementary material for: Prior resilience to trauma & coping during the COVID-19 pandemic
Source: PLoS One. 2024 May 7;19(5):e0297169. doi: 10.1371/journal.pone.0297169 (PMC11075842; doi:10.1371/journal.pone.0297169)
Supplement: S1 Table — (PDF) [file pone.0297169.s003.pdf]

S1 Table. Pearson's Correlation Coefficients among Resilience, Coping Styles and Coping Strategies.

	Resilience	Avoidant Style	Approach Style	Distraction	Substance Use	Positive Reframing	Humor	Religion	Emotional Support	Behavioral Disengagement	Active Coping
Resilience	1.00	**-0.39**	**0.07**	**-0.18**	**-0.14**	**0.13**	0.03	**0.06**	**0.04**	**-0.28**	-0.00
Avoidant Style	**-0.39**	1.00	**0.40**	**0.80**	**0.45**	**0.22**	**0.22**	**0.04**	**0.35**	**0.54**	**0.40**
Approach Style	**0.07**	**0.40**	1.00	**0.50**	**0.10**	**0.79**	**0.37**	**0.28**	**0.82**	**0.09**	**0.84**
Distraction	**-0.18**	**0.80**	**0.50**	1.00	**0.19**	**0.33**	**0.25**	**0.07**	**0.41**	**0.23**	**0.48**
Substance Use	**-0.14**	**0.45**	**0.10**	**0.19**	1.00	0.02	**0.08**	**-0.09**	**0.11**	**0.15**	**0.10**
Positive Reframing	**0.13**	**0.22**	**0.79**	**0.33**	**0.02**	1.00	**0.35**	**0.33**	**0.45**	0.01	**0.50**
Humor	0.03	**0.21**	**0.37**	**0.25**	**0.08**	**0.35**	1.00	0.03	**0.30**	**0.04**	**0.26**
Religion	**0.06**	**0.04**	**0.28**	**0.07**	**-0.09**	**0.33**	**0.03**	1.00	**0.17**	-0.01	**0.17**
Emotional Support	**0.04**	**0.35**	**0.82**	**0.41**	**0.11**	**0.45**	**0.30**	**0.17**	1.00	**0.10**	**0.55**
Behavioral Disengagement	**-0.28**	**0.54**	**0.09**	**0.23**	**0.15**	0.01	**0.04**	-0.01	**0.10**	1.00	**0.11**
Active Coping	-0.00	**0.41**	**0.84**	**0.48**	**0.10**	**0.50**	**0.26**	**0.17**	**0.55**	**0.11**	1.00

Note: p<0.0001 are **bolded**
