# Supplementary material for: Prior resilience to trauma & coping during the COVID-19 pandemic
Source: PLoS One. 2024 May 7;19(5):e0297169. doi: 10.1371/journal.pone.0297169 (PMC11075842; doi:10.1371/journal.pone.0297169)
Supplement: S2 Table — (PDF) [file pone.0297169.s004.pdf]

S2 Table. Linear Regression Models: Covariates Predicting Coping Styles and Strategies.

	Approach Style		Avoidant Style		Distraction		Substance Use		Behavioral Disengagement		Self-Blame		Emotional Support		Positive Reframing		Active Coping		Humor		Religion	
	ß	95% CI	ß	95% CI	ß	95% CI	ß	95% CI	ß	95% CI	ß	95% CI	ß	95% CI	ß	95% CI	ß	95% CI	ß	95% CI	ß	95% CI
Age	**-.03**	**-.05, -.01**	.01	-.00, .03	**.06**	**.04, .07**	**-.02**	**-.04, -.00**	-.00	-.02, .01	**-.03**	**-.05, -.01**	-.01	-.02, .01	**-.05**	**-.07, -.03**	-.01	-.03, .01	**-.03**	**-.05, -.01**	**-.03**	**-.05, -.01**
Non-white race	**-.13**	**-.24, -.03**	-0.07	-.17, .02	-.00	-.10, .10	**-.12**	**-.22, -.01**	.03	-.07, .13	**-.13**	**-.22, -.04**	**-.17**	**-.27, -.06**	-.06	-.16, .04	**-.11**	**-.21, -.01**	**-.15**	**-.25, -.05**	**.20**	**.10, .30**
Parent Education																						
Some College	**.09**	**.04, .13**	**.08**	**.04, .12**	**.08**	**.04, .12**	.04	-.01, .08	.03	-.01, .07	**.05**	**.01, .09**	**.09**	**.05, .14**	.04	-.00, .08	**.07**	**.03, .12**	**.08**	**.04, .12**	-.01	-.06, .03
College Plus	**.18**	**.14, .22**	0.16	.12, .20	**.12**	**.08, .16**	**.14**	**.10, .19**	**.05**	**.01, .09**	**.11**	**.07, .15**	**.20**	**.16, .24**	**.10**	**.06, .14**	**.14**	**.10, .19**	**.15**	**.11, .19**	**-.08**	**-.12, -.04**
Marital Status																						
Divorced/ Separated	-.05	-.11, .02	-.02	-.08, .04	-.02	-.09, .04	-.01	-.08, .06	-.04	-.11, .02	.01	-.05, .07	**-.13**	**-.20, -.06**	.01	-.06, .08	.00	-.07, .07	.05	-.02, .12	**-.11**	**-.18, -.04**
Widowed	-.05	-.14, .03	**-.11**	**-.18, -.03**	-.05	-.13, .03	-.07	-.16, .01	-.08	-.16, .00	**-.10**	**-.17, -.02**	**-.12**	**-.20, -.03**	.05	-.03, .14	-.07	-.15, .02	.01	-.07, .10	.06	-.02, .14
Single	**-.12**	**-.21, -.03**	-.09	-.18, -.01	-.07	-.16, .02	**-.12**	**-.21, -.03**	-.06	-.15, .03	-.03	-.12, .05	**-.14**	**-.23, -.05**	**-.10**	**-.19, -.01**	-.05	-.14, .04	.05	-.05, .14	**-.13**	**-.22, -.04**

Median Census Tract Income Quartile (2009)																						
Q1	-.04	-.09, .01	**-.15**	**-.20, -.11**	**-.17**	**-.21, -.12**	**-.15**	**-.20, -.10**	**-.05**	**-.10, -.01**	-.02	-.06, .03	**-.08**	**-.13, -.03**	**.07**	**.02, .12**	**-.08**	**-.13, -.03**	**-.09**	**-.14, -.04**	**.35**	**.31, .40**
Q2	-.00	-.05, .04	**-.09**	**-.13, -.04**	**-.10**	**-.14, -.05**	**-.12**	**-.17, -.07**	-.03	-.08, .02	.00	-.04, .05	**-.05**	**-.10, -.00**	**.05**	**.01, .10**	-.01	-.06, .04	-.05	-.10, .00	**.27**	**.23, .32**
Q3	.02	-.02, .07	-.04	-.09, .00	-.01	-.06, .03	**-.10**	**-.14, -.05**	-.03	-.07, .02	-.01	-.06, .03	.01	-.03, .06	**.08**	**.03, .13**	-.03	-.08, .02	-.03	-.08, .02	**.19**	**.14, .23**
Living alone	**.10**	**.03, .17**	**.11**	**.04, .17**	**.13**	**.07, .20**	.04	-.03, .11	.02	-.05, .08	.03	-.03, .09	**.09**	**.02, .16**	.04	-.03, .11	**.11**	**.04, .18**	-.05	-.12, .02	.02	-.05, .09
Active Healthcare Professional	**.05**	**.01, .09**	.03	-.00, .07	-.02	-.06, .02	**.06**	**.01, .10**	**.07**	**.02, .11**	**.04**	**.00, .08**	.01	-.03, .06	**.04**	**.00, .08**	**.06**	**.02, .10**	**.06**	**.02, .10**	.00	-.04, .04
Chronic Health Condition	-.03	-.07, .02	-.02	-.07, .02	-.01	-.06, .04	-.03	-.08, .02	-.01	-.05, .04	-.02	-.07, .02	-.01	-.06, .04	**-.05**	**-.10, -.01**	-.00	-.05, .05	.01	-.03, .06	-.01	-.06, .04

Note: p<0.05 are **bolded**
