# Supplementary material for: Prior resilience to trauma & coping during the COVID-19 pandemic
Source: PLoS One. 2024 May 7;19(5):e0297169. doi: 10.1371/journal.pone.0297169 (PMC11075842; doi:10.1371/journal.pone.0297169)
Supplement: S3 Table — (PDF) [file pone.0297169.s005.pdf]

S3 Table. Unadjusted Associations between Resilience and Coping Styles and Strategies.

	Pre-pandemic Resilience to Trauma		
	ß	SE	*p*
Coping Style			
Approach Coping	**0.05**	0.006	<0.0001
Avoidant Coping	**-0.18**	0.004	<0.0001
Coping Strategies			
Distraction	**-0.15**	0.007	<0.0001
Substance Use	**-0.09**	0.005	<0.0001
Behavioral Disengagement	**-0.20**	0.006	<0.0001
Self-Blame	**-0.26**	0.005	<0.0001
Emotional Support	**0.03**	0.008	<0.0001
Positive Reframing	**0.11**	0.007	<0.0001
Active Coping	-0.00	0.008	0.7254
Humor	**0.03**	0.008	<0.0001
Religion	**0.06**	0.009	<0.0001

Note: Coping and Resilience variables were standardized for interpretability.
